# Indoor Social Networks in a South African Township: Potential Contribution of Location to Tuberculosis Transmission

**DOI:** 10.1371/journal.pone.0039246

**Published:** 2012-06-29

**Authors:** Robin Wood, Kimberly Racow, Linda-Gail Bekker, Carl Morrow, Keren Middelkoop, Daniella Mark, Stephen D. Lawn

**Affiliations:** 1 Desmond Tutu HIV Centre, Institute of Infectious Diseases and Molecular Medicine, University of Cape Town, Cape Town, South Africa; 2 Department of Medicine, University of Cape Town Faculty of Health Sciences, Cape Town, South Africa; 3 Department of Science and Technology/National Research Foundation, Centre of Excellence in Epidemiological Modeling and Analysis, University of Stellenbosch, Cape Town, South Africa; 4 Department of Clinical Research, Faculty of Infectious and Tropical Diseases, London School of Hygiene and Tropical Medicine, London, United Kingdom; University of British Columbia, Canada

## Abstract

**Background:**

We hypothesized that in South Africa, with a generalized tuberculosis (TB) epidemic, TB infection is predominantly acquired indoors and transmission potential is determined by the number and duration of social contacts made in locations that are conducive to TB transmission. We therefore quantified time spent and contacts met in indoor locations and public transport by residents of a South African township with a very high TB burden.

**Methods:**

A diary-based community social mixing survey was performed in 2010. Randomly selected participants (n = 571) prospectively recorded numbers of contacts and time spent in specified locations over 24-hour periods. To better characterize age-related social networks, participants were stratified into ten 5-year age strata and locations were classified into 11 types.

**Results:**

Five location types (own-household, other-households, transport, crèche/school, and work) contributed 97.2% of total indoor time and 80.4% of total indoor contacts. Median time spent indoors was 19.1 hours/day (IQR:14.3–22.7), which was consistent across age strata. Median daily contacts increased from 16 (IQR:9–40) in 0–4 year-olds to 40 (IQR:18–60) in 15–19 year-olds and declined to 18 (IQR:10–41) in ≥45 year-olds. Mean daily own-household contacts was 8.8 (95%CI:8.2–9.4), which decreased with increasing age. Mean crèche/school contacts increased from 6.2/day (95%CI:2.7–9.7) in 0–4 year-olds to 28.1/day (95%CI:8.1–48.1) in 15–19 year-olds. Mean transport contacts increased from 4.9/day (95%CI:1.6–8.2) in 0–4 year-olds to 25.5/day (95%CI:12.1–38.9) in 25–29 year-olds.

**Conclusions:**

A limited number of location types contributed the majority of indoor social contacts in this community. Increasing numbers of social contacts occurred throughout childhood, adolescence, and young adulthood, predominantly in school and public transport. This rapid increase in non-home socialization parallels the increasing TB infection rates during childhood and young adulthood reported in this community. Further studies of the environmental conditions in schools and public transport, as potentially important locations for ongoing TB infection, are indicated.

## Introduction

Tuberculosis (TB) notification rates in South Africa have increased progressively over the past 20 years [1]. While the human immunodeficiency virus (HIV) epidemic has undoubtedly contributed significantly to the increased burden [1], [2], the underlying TB burden among the HIV-uninfected population has also remained extremely high [3]. Cape Town, a city of 3.4 million residents, notified almost 30,000 new cases of TB in 2009 to the national TB control program [4]. The TB notification rate within the HIV-uninfected population of Cape Town was extremely high at 386/100,000, with the largest proportions of new TB rates notified in young children (511/100,00) and young adults (553/100,000) [4]. The TB notification rates in the young HIV-negative Cape Town population are very similar to those recorded in early 20th century Europe prior to the implementation of effective chemotherapy [4], [5], [6].

The acquisition of TB infection in Cape Town is very high during childhood and adolescence, with childhood infection rates, determined by tuberculin skin testing, remaining at approximately 4% per annum, which has resulted in a TB infection prevalence of 20% in children starting school [7]–[10]. The annual rate of TB infection increases throughout childhood to 7% per annum during adolescence, resulting in a TB infection prevalence of 80% in young adulthood [11]. In order to maintain high TB infection rates in the general population, there must be intense exposure of the susceptible population to prevalent infectious TB cases.

In 2010, we conducted a social mixing study among a randomly selected sample of residents of a Cape Town African township [12]. The objective of the initial study was to use inter-personal contact information to model the potential for epidemic spread of an acute respiratory epidemic in a crowded township population. As we had previously reported high rates of TB disease and high annual risk of TB infection in children in this community [7], we went on to measure social mixing within indoor locations that might be driving transmission. We therefore determined the types of indoor locations frequented by the township residents, together with the numbers of contacts met and the time spent within each location. Contact information was then stratified by age and location in order to establish age-related social mixing patterns and postulate the potential impact on TB transmission.

## Methods

### Study Community

This study community is an African township, with approximately 18,000 residents of low socio-economic status who live in informal shacks and brick dwellings. The community is located 40km south of Cape Town and is highly affected by HIV and endemic TB [7], [8], [13]–[17]. Detailed descriptions of this community have been previously published [13]–[17].

### Social Mixing Survey Participant Selection

Participants were randomly selected in 5-year age strata from household census data. Residents who had emigrated were replaced with randomly selected participants from the same age stratum. Since the census was conducted 2 years prior to the study, women 15–45 years old and their children (0–4 years old) were randomly selected from the community. A detailed description of the selection process for this study has been previously described [12].

### Consent

The Human Research Ethics Committee of the University of Cape Town approved the study.

All participants provided written informed consent or assent. Parental/guardian written informed consent was also obtained for participants 12–17 years old. Participants received shopping vouchers valued at 75 South African Rand and community educators were incentivized to increase recruitment and retention of participants.

### Diary Survey

Participants underwent face-to-face diary completion training and completed a paper diary over a 24-hour period (5am–5am). Participants ≥12 years old completed diaries themselves. Parents/guardians of participants <12 years old were trained and assisted in completing the diaries. If a child was separated from a parent/guardian, another adult in charge at the specific location helped to complete the diary and provided feedback to the parent/guardian. Participants recorded information about the locations they visited during their daily routine, which included type of location, time of day, duration of time spent, and number of contacts met. Participants completed follow-up interview within 48 hours of diary completion to complete missing data and clarify inconsistencies.

### Data Analysis

Data were analyzed using STATA 11.0 (StataCorp, College Station, TX, USA). Midpoints between time-category limits were used to calculate the average contact time spent at each location. For example, “1–2 hours” was considered 1.5 hours and “more than 12 hours” was considered 18 hours. Descriptive statistics and linear regression models were employed to examine the associations of age strata with time spent and number of contacts in different locations. All statistical tests were 2-sided (α = 0.05).

## Results

### Diary Records

738 randomly selected residents were invited to participate in the study. Of these, 39 declined to participate, 10 withdrew, 4 were lost to follow-up and 8 did not return completed diaries. Concerns were raised about the rigor of one field worker’s data collection, and therefore to ensure the validity of our data, a decision was made to exclude a further 106 diaries. A detailed description of the sampling procedures for the study has been previously reported [15]. The remaining 571 participants completed diaries on the following days of the week: Sundays 11.6% (66 / 571), Mondays 9.8% (56 / 571), Tuesdays 13.1% (75 / 571), Wednesdays 23.6% (135 / 571), Thursdays 12.8% (73 / 571), Fridays 11.4% (65 / 571) and Saturdays 17.7% (101 / 571). Weekend activity was recorded in 29.2% (167 / 571) and weekday activities in 70.8% (404 / 571) of diary records

### Total Hours Spent and Number of Contacts Met in Indoor Locations

The total hours spent in indoor locations (10,765 hours) and the total number of indoor contacts met (n = 25,098) by 571 township residents during a 24-hour period stratified by age and location are shown in Tables S1 and S2. A graphical summary of these data across 11 locations is shown in Figure S1. Own household, other household, transport, crèche or school, and work locations accounted for 97.2% (10,460.2 / 10,765.0) of indoor time and 80.4% (20,175 / 25,098) of indoor contacts. The median daily time spent indoors per participant was 19.1 hours (IQR: 14.6–22.7). For every 1-year age increase, hours spent in indoor locations decreased by 0.002 (95%CI: −0.04 – −0.039), although this finding did not reach statistical significance (p = 0.90). The median number of daily contacts per participant was 30 (IQR: 12–54). However, the number of contacts varied markedly by age as shown in Figure S2. For every 1-year age increase (between the ages of 0–20 years), the number of contacts increased by 0.004 (95%CI: 0.002–0.006; p<0.001) and decreased by 0.006 (95%CI: −0.010 – −0.002; p = 0.004) for every 1-year age increase thereafter.

### Household Contacts

Total time spent in households contributed 25.1% (6,312 / 25,098) of indoor contacts and 84.9% (9,139.8 / 10,765.0) of indoor time. Own households contributed 80% (5,047 / 6,312) and other households contributed 20% (1,265 / 6,312) of total household contacts. The mean daily number of own household contacts was 8.8 (95%CI: 8.2–9.4). The mean daily number of household contacts stratified by age is shown in Figure S3A. Own household contacts decreased by 0.04 (95%CI: −0.070 – −0.003; p = 0.03) for every 1-year age increase, although other household contacts did not vary significantly by age (p = 0.45). The median number of own household contacts per encounter was 3 (IQR: 2–5), indicating a high degree of clustering.

### Crèche and School Contacts

The average classroom size of public schools in the Western Cape Province is 36 students [18]. Crèche/school contacts were reported by 19.7% (13 / 66) of children 0–4 years and 52.8% (102 / 193) of 5–19 year olds. Amongst 5–19 year olds, crèche/school contributed 42.3% (4,227 / 9,991) of indoor contacts and 11% (435.5 / 3,821.9) of indoor time. 92.3% (4,638 / 5,027) of contacts in crèche/school were met by 117 participants <20 years old, which resulted in a mean daily contact number of 39.6 (95%CI: 27.5–51.8) in this age group. The mean daily number of crèche/school contacts stratified by age is shown in Figure S3B. For every 1-year age increase in 0–19 year olds, contacts increased by 0.004 (95%CI: 0.001–0.007; p = 0.008). The median number of contacts per encounter were 29 (IQR: 18–32) and 33 (IQR: 25–40) in crèche and school respectively.

### Transport Contacts

In the Western Cape Province, 2/3 of the population use mini-bus taxis, trains, buses and other types of taxis respectively [19]. In this community, transport accounted for 27.2% (6,833 / 25,098) of indoor contacts and 2.3% (249.9 / 10,765.0) of indoor time. 52.1% (3,563 / 6,833) of transport contacts occurred in minibus taxis, 37.4% (2,554 / 6,833) in trains, 5.8% (393 / 6,833) in buses and 4.7% (323 / 6,833) in small vehicles. The mean daily number of transport contacts stratified by age is shown in Figure S3C. For every 1-year age increase in 0–29 year-olds, contacts increased rapidly by 0.01 (95%CI: 0.008–0.020; p<0.001). All age groups reported transport contacts, but 26.9% (1,836 / 6,833) were <20 years old. The median number of contacts per journey were 4 (IQR: 2–11), 15 (IQR: 14–16), 30 (IQR: 20–44) and 46 (IQR: 32–67) in small vehicles, minibus taxis, buses and trains respectively.

### Work Contacts

The unemployment rate in Cape Town townships is approximately 50% [20]. Work locations accounted for 8% (2,003 / 25,098) of indoor contacts and 4.9% (530.7 / 10,765.0) of indoor time. The mean daily number of work contacts stratified by age is shown in Figure S3D. 98.5% (1,973 / 2,003) of work contacts were ≥20 years old. The median number of work contacts per day was 9 (IQR: 4–27).

### Community Buildings, Shops, Shebeens, and Health Clinic Contacts

Community buildings contributed 6.0% (1,512 / 25,098) of indoor contacts and 0.7% (79.0 / 10765.0) and of indoor time. Community buildings, community halls, churches, and libraries accounted for 45.3% (685 / 1,512), 38.3% (579 / 1,512), and 16.4% (248 / 1512) of contacts respectively. The median number of contacts per visit were 18 (IQR: 9–45), 31 (IQR: 20–40) and 13 (IQR: 9–19) in community halls, churches and libraries respectively. Shops contributed 8.8% (2,222 / 25,098) of indoor contacts and 0.7% (76.9 / 10,765.0) of indoor time. Contact numbers varied markedly with type of shop; the median number of contacts per visit were 6 (IQR: 3–12) and 97 (IQR: 25–120) in local township shops and malls respectively. Shebeens (informal bars) contributed 2.9% (727 / 25,098) of indoor contacts and 0.7% (80.1 / 10,765.0) of indoor time. The median number of contacts per visit was 27 (IQR: 20–36). Health clinic contacts contributed 0.5% (128 / 25,098) of indoor contacts and 0.7% (80.1 / 10,765.0) of indoor time. The median number of health clinic contacts per visit was 23 (IQR: 8–32). “Other” locations along with indoor sporting activities only contributed 1.3% (334 / 25,098) of indoor contacts and 0.5% (56.9 / 10,765.0) of indoor time.

### Sputum Smear-positive TB Cases

The number and rate of smear-positive TB cases stratified by age in the study community from 1996–2011 is shown in Table S3. These data provide an indication of the prevalence of infectious TB cases in each age stratum able to contribute to transmission. Children 5–9 years old had the lowest smear-positive TB rate of 35 / 100,000 (relative rate ratio (RRR) = 0.10), which rapidly increased with age to 1692 / 100,000 (RRR = 1.00).

## Discussion

To our knowledge this is the first study to quantify time spent in indoor locations, which we hypothesized is where TB transmission is occurring within this community. Residents spent a large proportion of their time indoors and the proportion of time did not vary significantly across age strata. However, the types of locations visited and the number of contacts met in those locations varied markedly across age strata. The number of indoor social contacts met during childhood and adolescence steadily increased with advancing age. The increase in socialization during these ages may in part explain the increase in annual TB infection rate from 4% per annum during childhood to 7% per annum in adolescence, which has been previously reported in this community [7], [8].

Five main types of locations (own households, other households, school/crèche, work and transport) contributed the majority of indoor contacts and contact time. These “hotspots” may therefore offer the greatest potential for driving TB transmission in this community. The number of daily contacts for each age group was not normally distributed and the interquartile range was relatively wide, indicating that social activity within this community is very heterogeneous. Future studies might investigate whether those with high social contact numbers are at particularly increased risk of TB and other infectious diseases.

Participants of all ages reported transport use and the greatest number of contacts occurred while using transport, which provided the opportunity for inter-generational mixing - a pre-requisite for maintaining endemic TB. However, the time of exposure during transport was relatively short, which indicated that potential transmission of TB could be highly dependent on the infectiousness of TB cases and the quality of ventilation during journeys. The highest smear-positive TB rates were in adults, which illustrates that contacts between children/adolescents and adults in transport might have a large impact on transmission and sustaining endemic TB. Use of transport has been reported in a wide variety of settings to be associated with TB outbreaks [21]–[24]. The predominant forms of transport used by both children and adults in South Africa are informal minibus taxis and train services [19]. Minibus taxis have been documented to be extremely overcrowded and poorly maintained [25]. Increasing use of crowded transport during childhood and adolescence could therefore potentially contribute to the rapid acquisition of TB in these age groups.

In contrast to transport, household contact numbers were much lower, but the time spent in households was prolonged, which also provides a high potential for inter-generational mixing. Additionally, as households consist predominantly of family and friends, clustering amongst household residents and regular visitors is likely to be high. Therefore, there is a high probability of TB transmission from an infectious source to household members that, because of the long shared exposure time, would be less dependent on prevailing environmental conditions. Household transmission in this community has been documented to predominantly impact young children [17], which is consistent with the combination of long exposure times and intergenerational mixing within households. In contrast, adult TB disease transmission has been shown to be predominantly outside the household, which may result from increased participation in social networks [16].

In contrast to social mixing in transport and households, contacts made during crèche/school and work were highly age-assortative, which may impact the potential for TB transmission in particular age groups. Children 5–9 years old had the lowest smear-positive TB rates, which may limit potential child-to-child risk of TB transmission in crèche/school. However, smear-positive TB rates were 10-fold higher amongst 15–19 year-olds, which provides the potential for child-to-child transmission in this age group. Furthermore, high smear-positive rates in older adults highlight the potential for inter-generational TB transmission between teachers and students. The high smear-positive rates among adults highlight the implications for co-worker-to-co-worker TB transmission.

Non-household social contacts in shebeens, health clinics and churches, were previously identified as significant contributors to TB transmission in Cape Town [26]–[29]. However, in our quantitative study, the low number of contacts in shebeens and the infrequent presence of children or adolescents indicate that shebeens are unlikely to contribute significantly to the high TB infection rate observed during adolescence. Similarly, while the potential for transmission of drug resistant TB strains in health clinics necessitates the ongoing improvements of environmental conditions within health facilities, the very low contact numbers reported in this study indicate that health clinics contribute minimally to transmission. However, our study indicated that churches and other communal buildings have potential for TB transmission, especially from highly infectious TB sources.

There were limitations to our study. Potential information bias may have been introduced because parents/guardians completed diaries for children participants. However, to reduce recall bias, we used real-time data collection and conducted in-depth training and debriefing sessions with all participants and parents/guardians. In addition, 106 diaries submitted by one field worker were excluded; however, it is unlikely that selection bias was introduced given that each field worker was assigned a random selection of participants. It is also important to note that work, crèche/school, and health clinic social mixing data may be underreported given that nearly 1/3 of participants completed diaries during weekends.

The single identified location “hotspot” most capable of sustaining sufficiently high TB transmission within the general population was transport because all age groups used it, there was general mixing amongst passengers, and contact numbers were very high. Furthermore, overcrowding and poor ventilation in transport provide a very conducive environment for TB transmission. However, the other major locations may be individually unlikely to sustain TB transmission amongst the general population, as they involved small clusters of the population, not all age groups, and moderate contact numbers. Further studies of the prevailing indoor environmental conditions of frequented locations are required in order to definitively determine the TB transmission network.

In summary, we have identified a limited number of indoor location “hotspots” of social interaction with high potential for TB transmission. Increasing indoor socialization during adolescence parallels the reported increase in TB infection rates in this age group. The potential for household transmission is high but likely limited by clustering among small groups of individuals within each household. In contrast, the increasing use of crowded transport during adolescence with general population mixing and low levels of ventilation indicates that transport use offers high potential for maintaining TB transmission.

## Supporting Information

Figure S1 **Number of Total Contacts and Time Spent in 11 Indoor Locations.** The number of contacts and time spent at each indoor location is represented on a log scale and each Indoor Location is represented by a unique symbol. Own household, visited household, transport, crèche or school and work locations accounted for 97% of indoor-time and 80.4% of indoor contacts.
(TIF)

Figure S2 **Box and Whisker Plots of Total Indoor Contacts Stratified by Age.** The box and whisker plots represent the minimum, 25^th^ percentile, median, 75^th^ percentile, and maximum of total indoor contacts per age strata, which increased significantly for every 1-year increase in age between the ages of 0–20 (p<0.001) and decreased significantly for every 1-year increase in age thereafter (p = 0.004). Twenty-nine outliers were excluded from this figure to enhance visual clarity of the box and whisker plots but were not excluded from the statistical analysis.
(TIF)

Figure S3 **The mean number of daily contacts made per participant in households, crèche/school, transport, and work locations.** A. The mean number of daily own household [shaded bars] and other household [unshaded bars] contacts per participant with 95% confidence intervals for all age strata. B. The mean number of daily crèche and school contacts per participant with 95% confidence intervals for all age strata. C. The mean number of daily transport contacts per participant with 95% confidence intervals for all age strata. D. The mean number of daily work contacts per participant with 95% confidence intervals for all age strata.
(TIF)

Table S1 Hours spent indoors by 571 township residents during a 24 hour period, stratified by 5-year age strata and location. Number of participants, hours of indoor contact and mean hours of indoor contact across 11 different locations stratified by age are represented. Participants spent a total of 10,765 hours indoors during the total study period with a median of 19.1 hours per day (IQR: 14.6–22.7). Time spent indoors did not significantly vary across age strata (p = 0.90).
(XLSX)

Table S2 Number of indoor contacts of 571 township residents during a 24-hour period, stratified by 5-year age strata and location. Number of participants, number of indoor contacts and mean number of indoor contacts across 11 different locations stratified by age are represented. Participants met a total of 25,098 indoor contacts during the total study period with a median of 30 contacts per day (IQR: 12–54). For every 1-year increase in age between the ages of 0–20 years old, the number of contacts increased significantly (p<0.001) and decreased significantly for every 1-year increase in age thereafter (p = 0.004).
(XLSX)

Table S3 Age-stratified smear-positive TB cases in the study community, 1996–2011. Smear-positive TB cases and estimated population denominators were used to calculate smear-positive TB rates per 100,000 and Relative Rate Ratios (RRR), where the age stratum ≥45 was the reference category. Children 5–9 years old had the lowest smear-positive TB rate of 35 / 100,000 (RRR = 0.10), which rapidly increased with age to 1692 / 100,000 (RRR = 1.00).
(XLSX)
