# Supplementary figures and images for: Indoor Social Networks in a South African Township: Potential Contribution of Location to Tuberculosis Transmission
Source: PLoS One. 2012 Jun 29;7(6):e39246. doi: 10.1371/journal.pone.0039246 (PMC3387133; doi:10.1371/journal.pone.0039246)

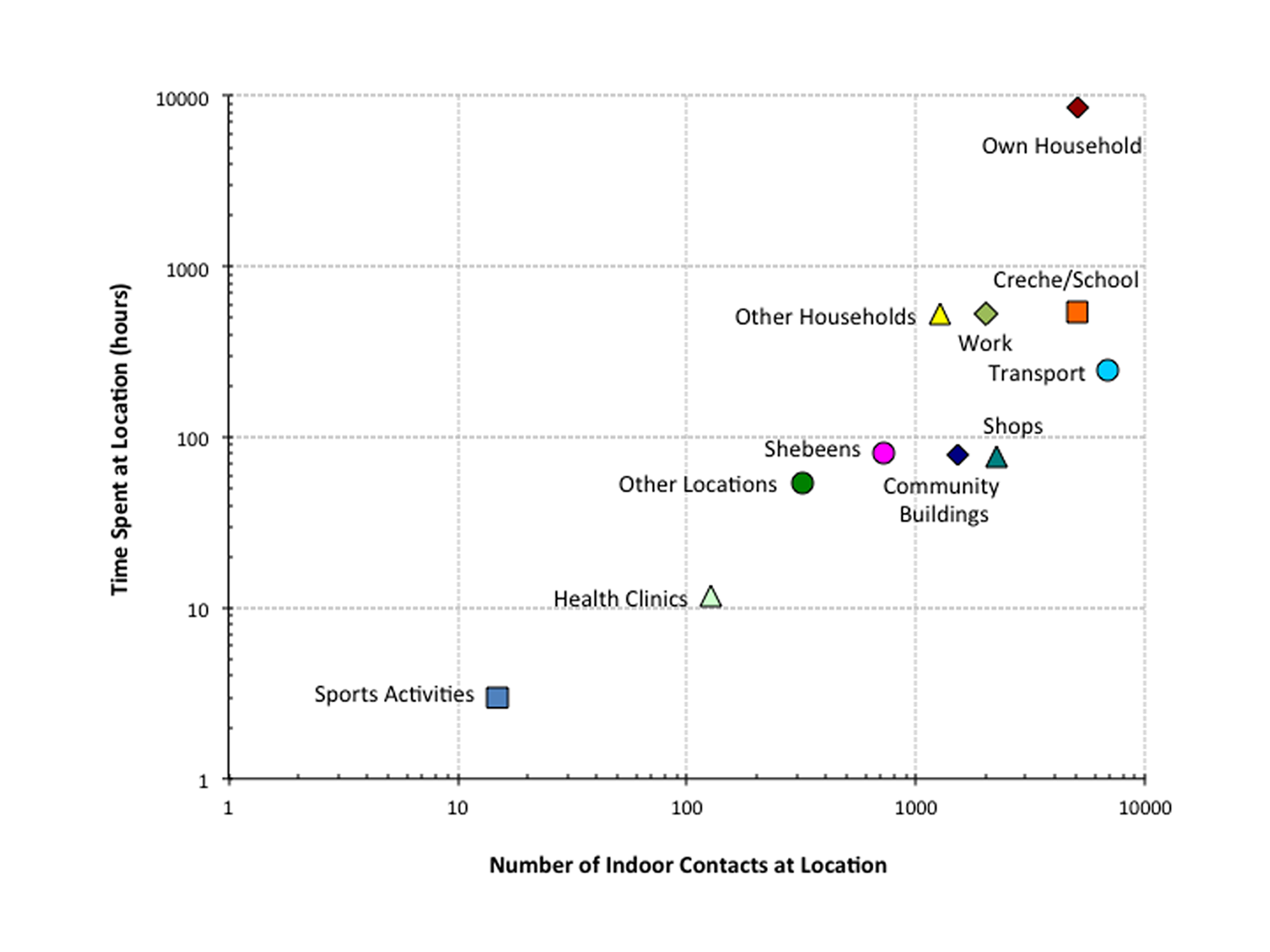

Supplement: Figure S1 — Number of Total Contacts and Time Spent in 11 Indoor Locations. The number of contacts and time spent at each indoor location is represented on a log scale and each Indoor Location is represented by a unique symbol. Own household, visited household, transport, crèche or school and work locations accounted for 97% of indoor-time and 80.4% of indoor contacts. (TIF) [file pone.0039246.s001.tif]

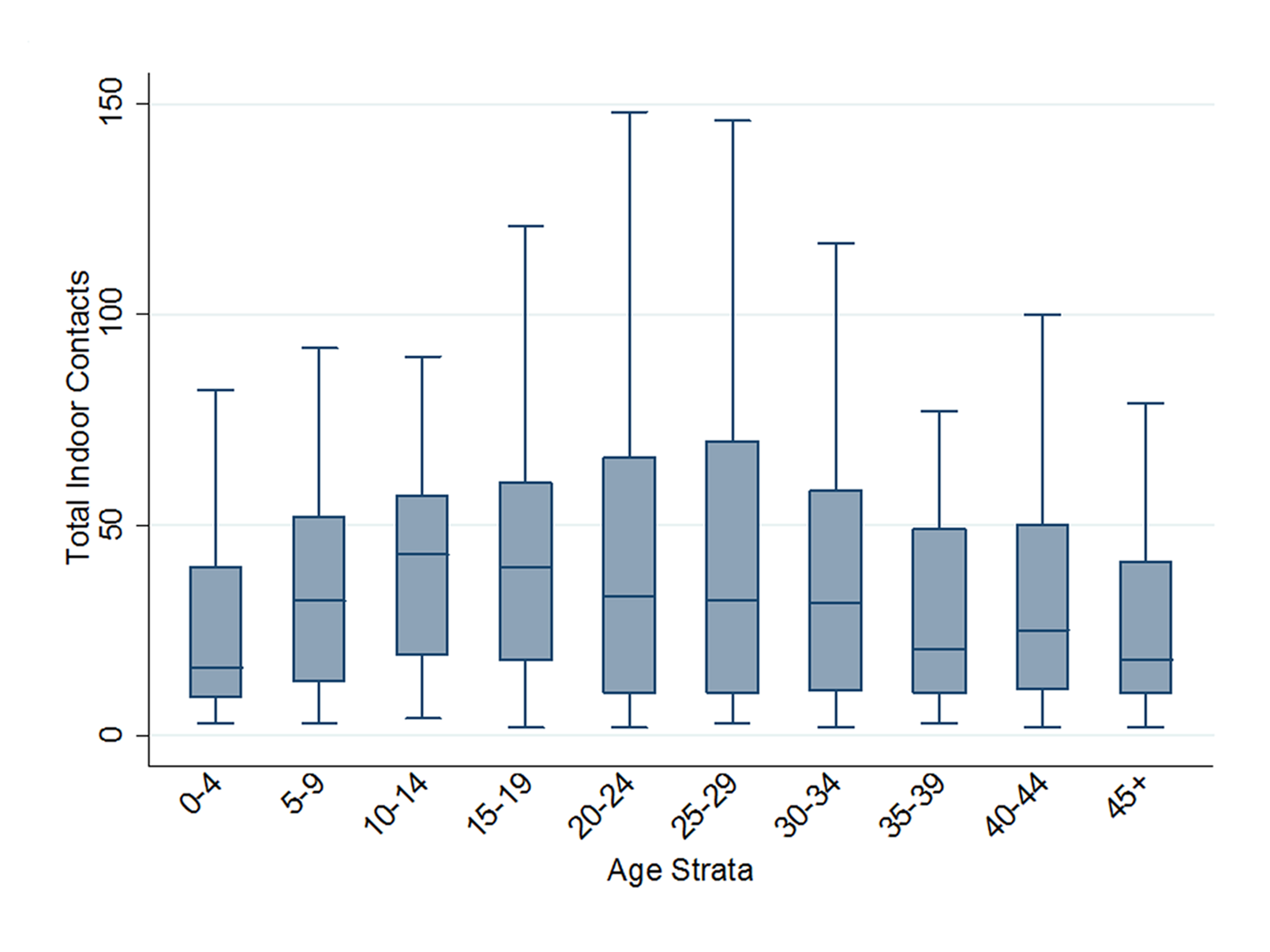

Supplement: Figure S2 — Box and Whisker Plots of Total Indoor Contacts Stratified by Age. The box and whisker plots represent the minimum, 25th percentile, median, 75th percentile, and maximum of total indoor contacts per age strata, which increased significantly for every 1-year increase in age between the ages of 0–20 (p<0.001) and decreased significantly for every 1-year increase in age thereafter (p = 0.004). Twenty-nine outliers were excluded from this figure to enhance visual clarity of the box and whisker plots but were not excluded from the statistical analysis. (TIF) [file pone.0039246.s002.tif]

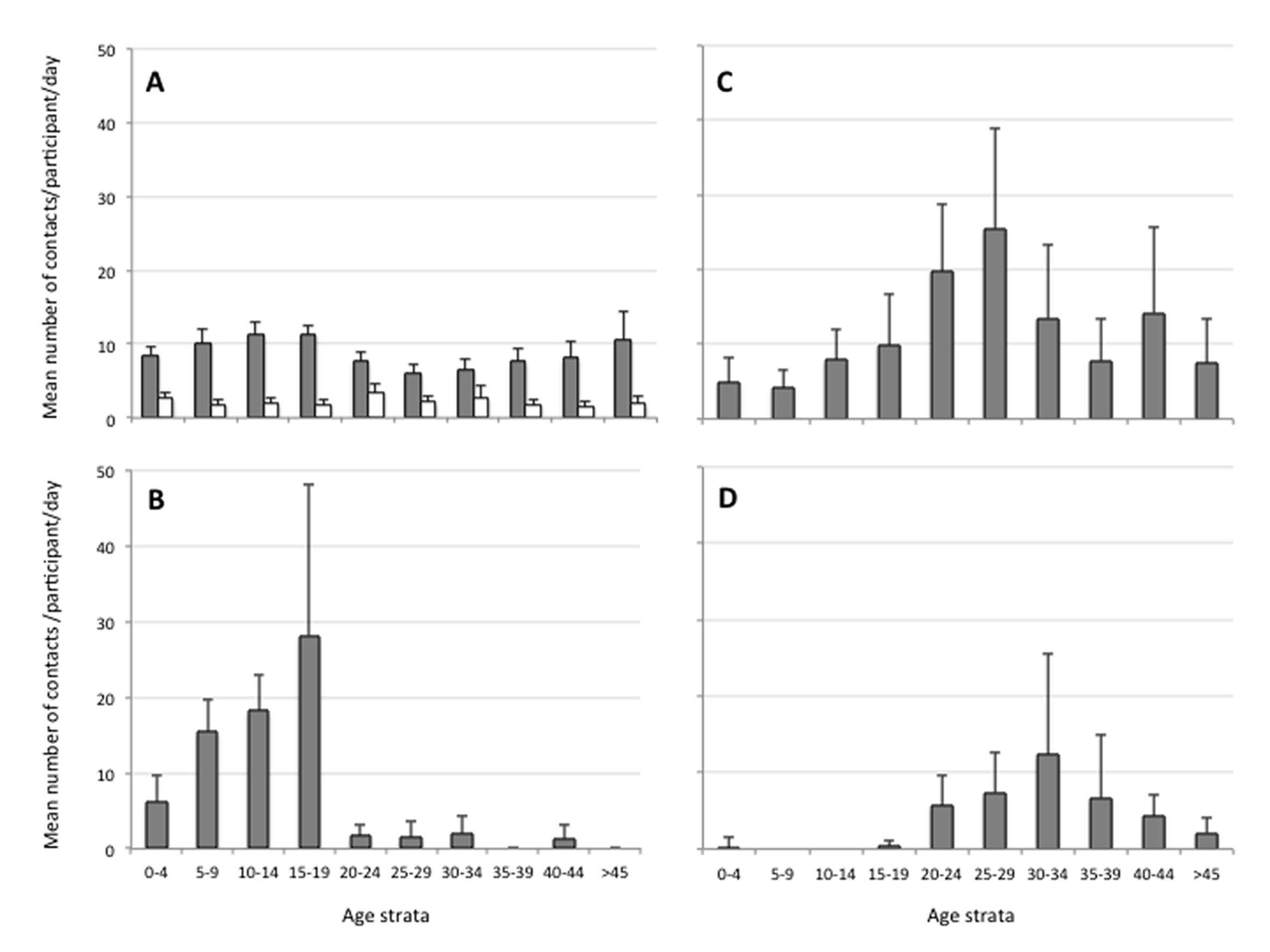

Supplement: Figure S3 — The mean number of daily contacts made per participant in households, crèche/school, transport, and work locations. A. The mean number of daily own household [shaded bars] and other household [unshaded bars] contacts per participant with 95% confidence intervals for all age strata. B. The mean number of daily crèche and school contacts per participant with 95% confidence intervals for all age strata. C. The mean number of daily transport contacts per participant with 95% confidence intervals for all age strata. D. The mean number of daily work contacts per participant with 95% confidence intervals for all age strata. (TIF) [file pone.0039246.s003.tif]
